# Systems Underlying Human and Old World Monkey Communication: One, Two, or Infinite

**DOI:** 10.3389/fpsyg.2019.01911

**Published:** 2019-09-03

**Authors:** Shigeru Miyagawa, Esther Clarke

**Affiliations:** ^1^Linguistics and Philosophy, Massachusetts Institute of Technology, Cambridge, MA, United States; ^2^Office of Open Learning, Massachusetts Institute of Technology, Cambridge, MA, United States; ^3^Behavioral Ecology and Evolution Research (BEER) Group, Durham University, Durham, United Kingdom

**Keywords:** language evolution, primate calls, call combinations, merge, Chomsky hierarchy

## Abstract

Using artificially synthesized stimuli, previous research has shown that cotton-top tamarin monkeys easily learn simple AB grammar sequences, but not the more complex A^n^B^n^ sequences that require hierarchical structure. Humans have no trouble learning A^n^B^n^ combinations. A more recent study, using similar artificially created stimuli, showed that there is a neuroanatomical difference in the brain between these two kinds of arrays. While the simpler AB sequences recruit the frontal operculum, the A^n^B^n^ array recruits the phylogenetically newer Broca’s area. We propose that on close inspection, reported vocal repertoires of Old World Monkeys show that these nonhuman primates are capable of calls that have two items in them, but never more than two. These are simple AB sequences, as predicted by previous research. In addition, we suggest the two-item call cannot be the result of a combinatorial operation that we see in human language, where the recursive operation of Merge allows for a potentially infinite array of structures. In our view, the two-item calls of nonhuman primates result from a dual-compartment frame into which each of the calls can fit without having to be combined by an operation such as Merge.

## Introduction

How did human language arise in evolution? To begin to answer this question, we must first decide what precisely we mean by language. Recently, Chomsky and others (Chomsky, 1995; Hauser et al., 2002) have proposed a characterization of language in which the core of the language faculty is composed of a computational system that contains one operation, Merge, which takes two syntactic objects and puts them together to form a set, {a, b}. For example, if *blue* is *a* and *book* is *b*, the output of Merge that operates on *a* and *b* would be {*blue, book*}. This output could in turn function as the input to another application of Merge, giving rise to the set {*the*, {*blue, book*}}. Much of syntax arises from this operation applying under a general requirement for computational efficiency, such as minimal search domain for Merge to combine *a* and *b*; this view of language is called the Strong Minimalist Thesis (SMT), and more recently, it has been referred to as the “Basic Property” of human language (Chomsky, 2000, 2013, 2016; Berwick et al., 2013; Berwick and Chomsky, 2016). As an example of computational efficiency, if {*blue, book*} serves as an input to Merge, the operation would select the closest object, which is the set itself, instead of prying into the inner structure of the set to pick *blue* or *book*. The recursive application of Merge gives rise to unbounded structured phrases, furnishing human language with the potential to generate an infinite array.

In contrast to the kind of view based on SMT, some scholars suggest that human language is primarily a culturally evolved system or a product of intensive gene-culture coevolution (Tomasello, 1996, 2000; Laland et al., 2000; Enfield and Levinson, 2006; Evans and Levinson, 2009; Chater and Christiansen, 2010; Azumagakito et al., 2018; Laland, 2018). According to this view, human language development relies predominantly on cultural learning skills, rather than on a set of categories predetermined by an innately-specified universal grammar, as Chomsky argues (Chomsky, 1980, 1981, 1988, 2007). We believe that there are aspects of language and evolution that would receive plausible explanation from a view that culture is central to the development and workings of language (e.g., the morphological variation we observe across languages). However, in this article, in which we will compare the basic workings of nonhuman primate and human systems underlying vocal communication, we believe that the SMT is the most appropriate theory of human language to use as a model against which to compare nonhuman primate alarm-calling systems. Other approaches include the theory that deconstructing language involves layers and degrees of complexity and therefore rejects a single structure-building operation such as Merge (Fitch, 2017; Townsend et al., 2018).

Often, scholars who adhere to the Merge + Computational efficiency view of language also suggest that the computational system that underlies language is unique to our species (Chomsky, 1968, 1980, 1981, 1988, 2007; Bolhuis et al., 2014). Note that this view of uniqueness is by no means entailed by the particular design of the computational system for human language; one could imagine other animals having a similar system, which complements recent assumptions (Townsend et al., 2018)^1^. The belief that the human language computational system is unique to humans stems from the observation that we do not find anything comparable to it in nonhuman primates or other animals (Hauser et al., 2002; Fitch and Hauser, 2004; Berwick et al., 2011; Schlenker et al., 2016b). This observation sometimes gives rise to the idea that what we find elsewhere in the animal world, such as the alarm calls of nonhuman primates, is so fundamentally distinct from human language that there are no meaningful commonalities between the systems (Smith and Kirby, 2008; Fischer, 2010). An argument often given in favor of the uniqueness of human language has to do with utility. One aspect of this is the notion that the typical nonhuman primate systems exist for the purpose of communication. For example, an alarm call for a particular predator is viewed as coextensive with the reference to that predator, and functions to communicate a message to or alter the behavior of others in the habitat regarding the predator, and/or to deter the predator itself (Maynard Smith, 1965; Zuberbühler et al., 1999a,b; Seyfarth and Cheney, 2003; Owren et al., 2010). In contrast, Zuberbühler et al. (1997, 1999a,b) provide experimental evidence based on the vocal behavior of Diana monkeys (*Cercopithecus diana diana*) that the calls are suggestively mediated by some form of cognitive semantic representations of the predator.

**Figure 1:**
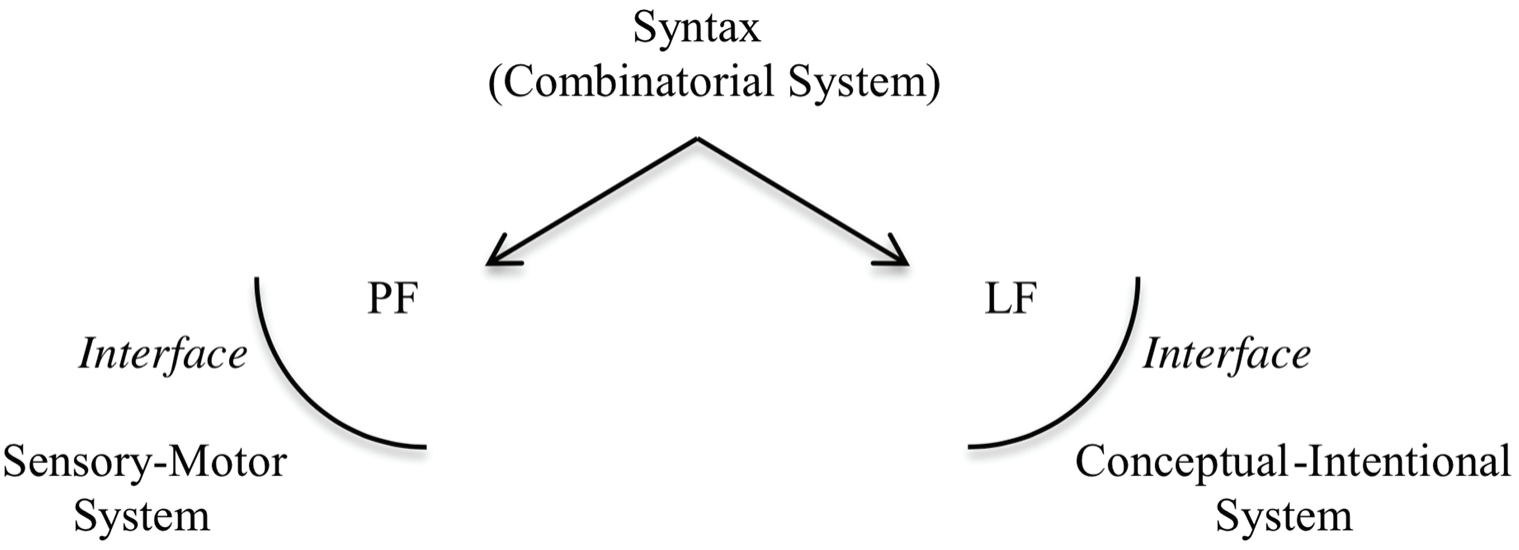
The architecture of the human Faculty of Language.

Human language has two components, the inner system, which is the computational system characterized by SMT, and the interfaces to which the array of structured phrases is sent: the phonological form interface (PF), which interacts with a sensory-motor system, associated with the externalization of the expressions generated; and the logical form interface (LF), which interacts with a conceptual-intentional system, responsible for interpretation. The architecture of the human language faculty, according to this view, roughly follows the representation in Figure 1.

The inner nature of the SMT computational system has led scholars to speculate that the utility of this system is not for communication but to represent thought (Chomsky, 2011, 2013; Berwick and Chomsky, 2016; Huybregts, 2017). As for the interfaces, setting aside LF, PF gives output to what we typically think of as language — the externalized form that is characteristically expressed by vocal means, although it could also be signs or written characters (Chomsky, 1995). In this way, sound (PF) and meaning (LF and its cognitive extensions) are only indirectly related, being mediated by the syntactic phrases generated by Merge. This may differ from primate alarm calls, which were originally characterized as having a direct link between the sound and its referent (Seyfarth et al., 1980b). However, further research shows this is by no means clear cut. There is evidence that acoustically distinct calls (a monkey alarm call and the corresponding predator vocalization) may elicit the same mental representation of the predator; thus, uncoupling the direct sound-referent link (Zuberbühler et al., 1999a,b). Similarly, context and other as yet unknown factors, may play a role in the iconic or symbolic nature of primate alarm calls (Fischer and Hammerschmidt, 2001; Price et al., 2015). For other relevant references, see, for example, Wich and de Vries (2006), Clay et al. (2015), and Scarantino and Clay (2014).

Despite the widespread belief that human language is unique to our species, with properties that are fundamentally different from systems found elsewhere in nature, a significant body of neuroscientific research on language has developed out of comparing human language with that of nonhuman primates. Fitch and Hauser (2004) showed that cotton-top tamarin monkeys are capable of learning the sequence (AB)^n^, which is based on a simple, regular grammar. But their ability to learn breaks down completely when exposed to the sequence A^n^B^n^, which is based on a formal grammar higher on the Chomsky Hierarchy (Chomsky, 1956) – what Fitch and Hauser term Phrase Structure Grammar, a combinatorial system that requires hierarchical relations that Merge would create in human language. Briefly, the experiment tested two groups of 10 tamarins, one for each grammar, on either a series of nonsense syllables with the simpler, (AB)^n^ sequence, for example, “no li pa ba” with alternative male and female voices for each syllable, or with the more complex A^n^B^n^ sequence, for example, “yo la pa do,” where the first two syllables were in the female voice and the last two in the male voice. A testing phase played back the following day, the same novel eight stimuli to both groups – four of which were consistent with (AB)^n^ and four of which were consistent with A^n^B^n^. About 72% of monkeys attended to violations of the (AB)^n^ sequences, but only 29% noticed violations to A^n^B^n^ sequences, suggesting the monkeys could only learn the simpler, finite state grammar sequences. In contrast, humans have no problem learning both types of sequences.

Using experimental stimuli modeled on Fitch and Hauser’s experiment, Friederici et al. (2006) showed that the more complex sequence, A^n^B^n^, activates the posterior portion of the Broca’s area (*viz*., Brodmann area 44) and also the frontal operculum. In contrast, the simpler sequence of (AB)^n^ only activates the frontal operculum. The frontal operculum is a phylogenetically older part of the brain compared to the Broca’s area (Sanides, 1962), and one of its functions is apparently to create sequences of (AB) combinations (Friederici et al., 2006), which we find both in monkeys (Sanides, 1962) and humans. On the other hand, the Broca’s area is a newer part of the brain compared to the frontal operculum. Studies have shown that each region has a unique functional, anatomical, and molecular brain architecture (Sanides, 1962; Amunts et al., 1999, 2010; Zilles and Amunts, 2009). For example, it is Broca’s region of the brain that is recruited for the more complex sequence-based Phrase Structure Grammar, which requires a hierarchical structure, and not the flat one we see for AB. Given that Merge^2^ is responsible for creating hierarchical structures, it is possible to view the Broca’s area as giving human language its distinct uniqueness by furnishing this operation to generate structured hierarchical arrays (Zaccarella and Friederici, 2015). Nevertheless, we acknowledge that other studies implicate the left anterior temporal lobe in human language combinatorial/hierarchical operations without mention of Broca’s area and the frontal operculum (Bemis and Pylkkänen, 2011; Brennan and Pylkkänen, 2017). The field of human brain research remains contentious and a discussion of the various viewpoints is beyond the scope of this article. Instead, we focus on the comparative human and nonhuman primate ability to combine call/word units and rely on studies that investigate these phenomena.

We wish to pursue a question parallel to Fitch and Hauser (2004), Friederici et al. (2006), namely, what is the difference between human and nonhuman primate systems that underlie communication? We will closely look at the research on Old World monkeys such as the Diana monkeys, Campbell’s monkeys, and De Brazza’s monkeys, to see what their vocal behavior can tell us about the actual system that underlies the primate communication system. It is typically believed that alarm calls, which are one stereotypical verbal behavior of monkeys, are composed of acoustically distinct, isolated utterances of alarm, such as those calls given in response to leopard, eagle, and snake predators (Blumstein, 1999). They do not combine, for example, the calls they give to leopards and eagles to create a novel utterance. However, research on the Old World monkeys indicates that some species have what appears to be a combinatorial system in which they can put together two independent items^3^. What we will show, based on the analysis of the reported data, is that these monkeys indeed have a way to create a two-term expression. This is consistent with Fitch and Hauser’s finding that tamarins can learn AB sequences (Fitch and Hauser, 2004). Assuming this AB sequence to be associated with the frontal operculum, this is also consistent with the observation that the frontal operculum supports the combining of two elements in sequence, rather than building a hierarchical structure (Zaccarella and Friederici, 2015). In the systems utilized by monkeys, we will see a specific way in which two elements can be put together.

Looking across the systems underlying communication in human and nonhuman primates, we observe that there are essentially three systems: one, two, and infinite. “One” refers to the well-known isolated alarm calls found across the primate world, especially observed in the alarm-calling system of vervet monkeys, while “infinite” refers to the infinite potential of the human language that is made possible by the recursive application of Merge. It is “two” that we will look at carefully; we will see that it is not based on any combinatorial system such as Merge, a point consistent with previous research. The question is, how is “two” made possible? The answer to this may hold a key to how Merge emerged in *Homo sapiens*.

We begin with a brief discussion of the “one” system.

## System of One

Several species of both Old and New World primates have what we call here “isolated” alarm calls, meaning one stereotyped utterance elicited by a specific predator/threat in the environment. Examples include the now famous vervet monkey system, studied first by Struhsaker (1967), and then, in more detail, by Seyfarth et al. (1980b). Vervet monkeys (*Chlorocebus pygerythrus*) give a distinct call when they see a leopard (“bark”), another when they see an eagle (“cough”), and a third when they encounter a snake (“chutter”). All three predators require distinct escape strategies and these calls, when experimentally played back to vervet groups, reliably elicit the appropriate reactions, even in the absence of the predator referent (Seyfarth et al., 1980a). Thus, scholars have concluded that these types of alarm calls should be classified as “functionally referential” (Macedonia and Evans, 1993) functioning as if they carry referential “meaning” to other vervets. Similarly, tamarins (*Saguinus fuscicollis* and *Saguinus mystax*) have an aerial alarm call and a distinct terrestrial alarm call, which both elicit appropriate anti-predatory behaviors (Kirchhof and Hammerschmidt, 2006). In both these cases, the alarm calls to different threat classes (aerial/terrestrial) or predators (eagle/snake/leopard) are acoustically distinct and are not combined to create calls relating to new referents or to carry new “meanings,” as far as we are aware. It is of note that the vervet monkey system, which has recently been revisited (Price et al., 2015), shows some intergradation between alarm calls and suggests contextual information, as well as pertinent acoustic cues, is important in determining a monkey’s behavioral response to alarm situations. Rather than absolutely discrete calls, these and probably other primates, are able to use similar call types more flexibly.

Functionally referential calls are not restricted to nonhuman primates in the animal kingdom. There are also at least six species of bird that use predator-specific alarm calls: Fowl, White-browned scrub wren, Siberian jay, Great tit, American robin, and Yellow warbler (reviewed in Gill and Bierema, 2013). Additionally, there are other mammals that use functionally referential calls, for example, Gunnison’s prairie dogs and domestic dogs (reviewed in Townsend and Manser, 2013). This suggests that the isolated alarm call may be much older than the direct ancestor of modern primates, or it may have evolved more than once in evolutionary history.

Despite an apparent lack of combinatory alarm calls, many nonhuman primates exhibit regular variation *within* isolated call types that may be used to convey different “meanings.” For example, red-fronted lemurs (*Eulemur fulvus rufus*) also rely on two alarm calls: a functionally referential call for aerial predators and a more generalized call for terrestrial predators and other ground disturbances. However, they vary the frequency and amplitude of their generalized terrestrial “woof” alarm call. This variation corresponds to threat urgency, with experimentally increased frequency and amplitude eliciting a higher arousal state (Fichtel and Hammerschmidt, 2002). Among the apes, evidence for referential alarm calls is surprisingly sparse. However, chimpanzees (*Pan troglodytes*) produce different *types* or *grades* of “rough grunt” that allow listening conspecifics to determine which type of food has been discovered (Slocombe and Zuberbühler, 2005). In one study, apples (a low value food) elicited a rough grunt with low fundamental frequency, whereas bread (a high value food) elicited a rough grunt with high fundamental frequency (among other varying acoustic parameters). Acoustic differences between the two rough grunts were statistically significant. Gibbons (*Hylobates lar*) also have graded calls, known collectively as “hoos,” which subtly vary in context-specific ways (Clarke et al., 2015). In both cases, imposed acoustic variation increases the utility of an isolated call and subsequently the vocal repertoire of the primates. Combining calls to form new meanings would increase the repertoire further, yet in many species evidence of this is lacking [chimpanzee pant-hoots may represent an example of a combined call but there is no evidence, as yet, that the constituent calls have independent “meanings” or that the entire sequence has a compound or new meaning (Zuberbühler, 2018)]. The point is that primate call systems exist that do not combine call elements in order to convey changes in call meaning, thus potentially explaining the dearth of call combinations and subsequent lack of Merge found in many nonhuman primate systems.

## System of Two

If the system underlying nonhuman primate communication does not contain Merge, as suggested in the work of Fitch and Hauser (2004) and others, a natural conclusion to draw is that the system associated with these primates cannot combine elements but are limited to the System of One with only isolated calls. However, there is a body of research on Old World monkeys, particularly the Guenons (*Cercopithecus*) of Africa, that indicates that these monkeys are capable of vocal behavior in which two elements are combined to form a third call that has “meaning” distinct from its parts. Human language contains at least two combinatorial systems (a duality of patterning) – a simple phonological system and a compositional, semantic system. The crucial difference is that in the compositional system, combined elements have compound meanings, derived from their constituent elements and the way in which they are combined, whereas in the phonological system this is not the case. In language, combined elements can be inserted into other sequences (recursion) and according to Merge theory, only Merge can account for these hierarchical structures. Does the system underlying the communication of these Old World nonhuman primates contain something resembling Merge, contrary to prior research? We do not believe so. The crucial fact, as far as we can determine, is that in every case, the combination is limited to two elements. One never finds a call made up of three or more parts to the call. What we suggest is that the system used by these monkeys contains a dual-compartment frame that allows them to acquire a two-part call. The two-part call is not the result of some combinatorial operation such as Merge, but rather, the nonhuman primate possesses this dual-compartment frame for creating utterances. Based on prior research, we speculate that this dual-compartment frame is the basis for nonhuman primates being able to learn AB sequences easily (Fitch and Hauser, 2004). Friederici et al. (2006)’s study suggests that the dual-compartment frame exists in the older part of the brain, in the frontal operculum, to allow nonhuman primates to learn AB sequences without the need of Merge, which in humans is in the Broca’s area^4^.

If the kind of analysis we are proposing for nonhuman primate and human systems underlying communication is correct, it adds to the debate about the origin of human language. In particular, there are scholars who advocate that human language developed through a series of protolanguages, from one-word, to two-words, and so on (e.g., Bickerton, 1990, 1998; Jackendoff, 1999, 2002). In our view, there was a sharp cut-off between the two-word stage and the kind of system we find in modern language that has the potential to generate an infinite array of structured phrases. Our ancestors, prior to developing Merge, simply recruited the same systems of one and two items that had developed in nonhuman primates. In principle, at this point, there was no difference between nonhuman and human vocal behavior. Once Merge developed, an entirely new system emerged that can recursively combine elements into an unbounded array of structured phrases, something we do not see in the nonhuman primate world. The only part of this new system that may have been inherited from the earlier system is binarity. It is well established that the structure of human language is binary (Kayne, 1984; Nowak et al., 2002; Toyota, 2012), and this property naturally arises from Merge that always combines two items. But why does Merge not combine three or more items? In principle, there is no reason why a combinatorial operation that creates a set of three {a, b, c} or more cannot be conceived. But we do not find this in human language, except possibly in highly special constructions such as conjunction. One possibility for the binary nature of human language comes from the dual-compartment frame that first developed in nonhuman primates. In this view, Merge emerged independently, but its input was furnished by the dual-compartment frame of the older system. This may relate to an idea that Friederici proposes (Friederici, 2004, 2009; Friederici et al., 2006) that the Broca’s area is involved in the processing of complex (hierarchical) syntax, while local syntactic structure building recruits the deep frontal operculum (see also Zaccarella and Friederici, 2015). In our analysis, the “local syntactic structure building” would be based on the dual-compartment frame, whereas Merge in the Broca’s area is responsible for complex syntax building^5^.

In an earlier work, Progovac (2015) proposes what she calls a two-slot mold, primarily to account for certain kinds of two-word compounds, two-word sentences, and paratactic attachment of two clauses such as *monkey see, monkey do*, which she considers as reflecting a primitive stage of human language. While we do not consider any combinations in modern human language to be “living fossils” of an older era (Nobrega and Miyagawa, 2015), we acknowledge that Progovac earlier proposed the idea of the two-term frame as a “proto” stage of human language, an idea compatible with our dual-compartment frame for monkeys.

It is worth noting here that nonhuman primate vocal systems may contain more call combinations than currently recognized. For example, some primate examples of the System of One may, on closer inspection, utilize a System of Two. One instance of this comes from the black-fronted titi monkey (*Callicebus nigrifrons*). A study published in 2012 showed that call A is given reliably to threats in the canopy, whereas call B is given to threats on the ground, and these calls are functionally referential (System of One) (Cäsar and Zuberbühler, 2012). A follow up study published in 2013 showed that these monkeys combine A and B calls (in predator-specific ways) to signify, for example, an aerial predator on the ground or a terrestrial predator in the canopy (System of Two) (Casar et al., 2013). An even closer look at the same titi monkeys’ combinations of A and B calls by Berthet et al. (2019) reveals more complexity. While the predator type seems more important than its location, both are revealed in the call combinations, particularly by the proportion of “BB-grams” (the proportion of two contiguous B calls). The authors suggest that the information is continuous rather than categorical and has elements of probabilistic meaning. In terms of our theory, the BB-grams would take up one slot (B^n^) in the dual compartment frame and the other would be taken up by the A calls (A^n^), still fulfilling the System of Two requirements. However, this example illustrates how the flexibility of monkey call combinations can still be expressed *via* the dual compartment frame theory. Further research is needed to shed light on how other monkeys produce and attend to their call combinations.

## Analyses of Old World Monkey Calls

We begin our analyses with the putty-nosed monkey (*Cercopithecus nictitans*), where we develop the idea of the dual-compartment frame for nonhuman primates. We will then apply this to some other Old World monkeys that also evidence a two-term combination.

### Putty-Nosed Monkey

Putty-nosed monkeys have two main alarm calls, *pyow*s (=P), which are broadly distributed and suggestive of a general alarm call, and *hack*s (=H), which are often used to indicate eagles (Arnold and Zuberbühler, 2012). In addition, the putty-nosed monkeys sometimes produce *pyow-hack* sequences composed of a small number of *pyow*s followed by a small number of *hacks.* Unlike the individual *pyow*s and *hacks*, which are alarm calls made in response to a perceived predator, the *pyow-hack* sequences are apparently predictive of group movement. The length of the sequence is statistically related to the distance traveled. In a series of playback experiments, Arnold and Zuberbühler (2006a,b, 2008, 2012, 2013) showed that it is the length of the overall sequence that is predictive of the distance traveled, and the actual composition of the equal-length sequences did not appear to affect the behavior. Thus, comparative behavioral results were obtained when PPPHHH, PHHHHH, and other P-H combinations of the same length were played back.

**Figure 2:**
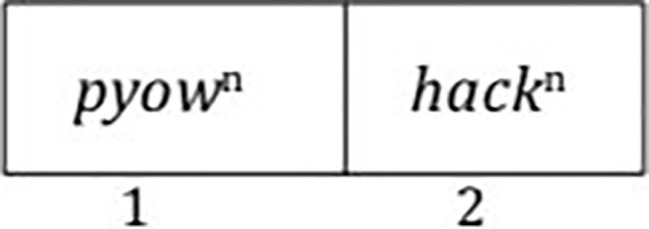
Dual-compartment frame proposed for putty-nosed monkey alarm calls.

What we see here, as Schlenker et al. (2016a) notes, is that the various *pyow-hack* sequences of the same length are phonologically complex, but lexically simple. They are phonologically complex because of the multitude of possibilities for the occurrence of *pyows* and *hacks*. But the sequence is lexically simple because regardless of the actual number of *pyows* and *hacks*, the sequence is apparently associated with comparable behavior — the distance traveled is essentially the same. How can we capture both the phonological complexity and the lexical simplicity of these sequences? When one looks at the various possibilities, there are two compartments, one for *pyows* and the other for *hacks* (Figure 2).

Within each compartment, one can have a varying number of *pyows* and a varying number of *hacks*. Crucially, one never finds a sequence that alternates between the two, such as PHPH… (Arnold and Zuberbühler, 2012). We would not expect such an alternation because it would require more than two compartments. So the *pyow-hack* sequence must always fit into a dual-compartment paradigm, with the only variable being the length of the overall sequence as dictated by the number of *pyows* and *hacks*. We suggest that this dual-compartment frame, which Progovac (2015) earlier proposed as “two-slot mold” for an ostensible human protolanguage, is responsible for what roughly appears to be a combinatorial process of word building in these monkeys. Crucially, there is no operator that operates on each term and combines them, as would be the case if Merge were available. This is clearly seen by the varying numbers of *pyows* and *hacks* that, despite the variation, form a unified expression with the same “meaning.” If some combinatorial operation were involved, we would need to say that this operation would take each instance of *pyow* and each instance of *hack* and combine them into some expression, but it is not clear what the structure of such an expression would be, nor is it clear how such combinatorial operations could predict that the overall meaning is the same regardless of the number of individual items in the call.

### Campbell’s Monkey

Ouattara et al. (2009b) reports on a study of adult males of six wild groups in the Tai Forest of Cote d’Ivoire. A striking property of the alarm calls of these monkeys is what Ouattara et al. call affixation, where an acoustically invariable “suffix” attaches to acoustically variable “stems.” Let us start by looking at the alarm calls of Campbell’s monkeys (*Cercopithecus campbelli*) (Table 1).

**Table 1:** Alarm calls of Campbell’s monkeys. Adapted from data in Ouattara et al. (2009b).

Call	Context
*boom*	Given in non-predatory cases, such as a falling branch
*hok*	Given when a crowned eagle is detected
*krak*	Given when detecting a leopard
*hok-oo*	Given to disturbances in the canopy, hence a general aerial call
*krak-oo*	Given to almost any disturbance
*wak-oo*	Given to the same events as *hok-oo* calls (eagles, etc.)

We will focus on four of these calls, *krak*, *hok*, and their “affixed” versions, *krak-oo* and *hok-oo*. Ouattara et al. (2009b), see also (Ouattara et al., 2009a; Schlenker et al., 2016a), note that the “affix” *oo* attaches to a stem to “broaden the call’s meaning.” In the case of *hok-oo*, the stem *hok* is a specific eagle alarm, while the affixed version is a general arboreal disturbance call. For *krak-oo*, the stem *krak* is a leopard alarm call while the affixed version is a general alert call. By calling *oo* an “affix,” Ouattara et al. (2009b) as well as Schlenker et al. (2016a) implicitly assume an operation by which *oo* is attached to a stem with some predictable semantic effect (see Schlenker et al., 2016a for a detailed semantic/pragmatic analysis, including dialects of Campbell’s monkey calls).

However, an equally plausible way to view these alarm calls is that they are learned as independent, whole calls, and the phonological and semantic resemblances we see with *oo* are entirely accidental. This would be consistent with the idea that Merge does not exist in the system underlying monkey communication, and is supported by data showing that the *oo* affix is produced as an independent articulation rather than a co-articulation (Kuhn et al., 2018). So which is it? Does Merge or some such operation exist in Campbell’s monkey system to operate on a stem and affix and combine them, or are these alarms simply learned as they are without any composition involved? We will carefully sift through the data (Ouattara et al., 2009b) in order to show that the Campbell’s monkey seems to be aware that in the call *krak-oo*, *krak* stands for leopard despite the fact that the overall call, *krak-oo*, is a general alarm call. But this does not entail the existence of a combinatorial operation such as Merge; we will argue when we look at the developmental data of De Brazza’s monkeys (*Cercopithecus neglectus*), which has a similar system as Campbell’s monkeys, that the calls appear to be learned as whole expressions even when there appears to be an affix, but at the same time, the monkey seems aware that there are parts of calls that carry meaning independent of the entire call. This way of looking at the “affixed” calls parallels what we saw for putty-nosed monkeys. The system that we identified for these monkeys has a dual-compartment frame, with each slot being populated by one or more of the same call, *pyow* or *hack*.

In order to show that Campbell’s monkeys are aware that *krak-oo* contains *krak* that signifies a leopard, we need to carefully sift through Ouattara et al.’s data (Ouattara et al., 2009b), and extract from it data that is most widely distributed among the population studied. In one experiment, the researchers presented both visual (model) and acoustic cues of eagle and leopard to the monkeys in their natural habitat. Focusing on the alarms elicited by the visual cue first, we find the following (Table 2).

**Table 2:** Number of call responses to visual predators by Campbell’s monkeys. Adapted from data in Ouattara et al. (2009b).

	krak-oo	krak	hok-oo	hok
Eagle_visual_	91		37	151
Leopard_visual_	4	273		

For eagle, the call specific to eagles, *hok*, was most numerous, but there were also *hok-oo*, which is a general arboreal call, and *krak-oo*, which is a general call. For the leopard visual cue, *krak,* which is the leopard call, is the call given. There were four *krak-oo* calls, given by just one of the seven animals, whereas the other calls were distributed across virtually all of the animals under study. We therefore believe that these four *krak-oo* calls are atypical and can be excluded, so that what we have is the following (Table 3).

**Table 3:** Number of call responses to visual predators by Campbell’s monkeys, excluding possible outliers.

	krak-oo	krak	hok-oo	hok
Eagle_visual_	91		37	151
Leopard_visual_	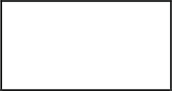	273		

As shown by the rectangle, there is a gap in the paradigm. Why did not all seven animals give out *krak-oo* when presented with a leopard when this call is a general call that would be appropriate for this context? We can see that for eagle, the monkeys gave out this general call in large numbers. A plausible explanation lies in the fact that *krak-oo* contains the form *krak*, which is the leopard alarm call. When faced with a leopard, the monkeys overwhelmingly chose to use *krak* instead of *krak-oo* because *krak-oo*, despite being a general call, nevertheless contains *krak* and apparently a residue of the meaning of leopard associated with it. Faced with a leopard in the vicinity, the Campbell’s monkey chose the more direct way to convey the alarm by choosing *krak* instead of *krak-oo*.

According to Schlenker et al. (2016a,b), the Informativity Principle is: “when one call is strictly more informative than another, the most informative one is used whenever possible” (p. 18). We can adapt and apply this to the Campbell’s monkey call system to get: “when two alarm calls contain reference to the same predator, the more informative one is used whenever possible.” The fact that this principle excludes *krak-oo* when presented with a leopard model suggests that the Campbell’s monkeys are aware that this expression is composed of two parts (and was not learned as an unanalyzable unit). This also explains why, when presented with an eagle model, the Campbell’s monkeys used both *hok*-, for eagle, and *krak-oo*, the general call; the latter does not contain any reference to the eagle, so it is not excluded by the revised Informativity Principle. There is a question as to why the Campbell’s monkeys also produced *hok-oo* when presented with the eagle model. This should be excluded by the Informativity Principle in favor of *hok*-. One possible explanation lies in the observation that *hok-oo* appears to have additional functions beyond *hok*- and is associated with distinctive behavior: “[w]hile producing “hok-oo” calls, males adopted a threat posture, combined with flashing their eyelids, and they sometimes conducted a short dash toward the disturbance” (Ouattara et al., 2009b:3).

The question still remains as to how the Campbell’s monkey learns *krak-oo*. Is it by affixation, as previous research suggests, or is it learned as a whole expression, but fitting into the dual-compartment frame as we saw for the system entailed for the putty-nosed monkey? The data available for Campbell’s monkeys do not help us to decide, but when we look at De Brazza’s monkey system (described later), which has calls similar to that of Campbell’s monkeys, we find evidence that there is no combinatorial operation involved during development, but rather, the two items in a call fit into a dual-compartment frame.

If we look now at the Campbell’s monkey calls elicited by acoustic cues, we get a very different result (Table 4).

**Table 4:** Number of call responses to acoustic predator cues by Campbell’s monkeys. Adapted from data in Ouattara et al. (2009b).

	krak-oo	krak	hok-oo	hok
Eagle_acoustic_	62		7 (3/7)	9 (2/7)
Leopard_acoustic_	67	42 (4/7)		

Let us exclude the two small instances, seven for *hok-oo*, which were elicited by just three out of the seven animals, and nine for *hok*, elicited from just two of seven animals. In addition, the 42 instances of *krak* were elicited from four out of seven animals, and of these four animals, two of them were responsible for 33 calls, or close to 80% of the total number of *krak* calls. If we temporarily exclude these 42 instances, we get the following (Table 5).

**Table 5:** Number of call responses to acoustic predator cues by Campbell’s monkeys, excluding possible outliers.

	krak-oo	krak	hok-oo	hok
Eagle_acoustic_	62			
Leopard_acoustic_	67			

What we can see is that contrary to the visual cues, the monkeys reacted to acoustic cues with uncertainty, thus they consistently and overwhelmingly used the most general alarm call regardless of the acoustic cue they heard. One explanation is that acoustic playbacks may be weaker experimental stimuli than visual models due to them being short-lived, and impossible to confirm, especially if a function of alarm calling is to deter the predator (Arnold et al., 2008). Thus acoustic predator cues may make for uncertain/non-uniform responses. Another possibility is that a vocalizing predator is unlikely to be hunting, and therefore does not represent as great a threat as a silent, but visualized predator. For most of the population, then, using the direct call, such as *hok* for eagle and *krak* for leopard, requires visual witnessing of the predator. The exception to this were the two animals that elicited a large number of *krak* calls in response to the acoustic leopard cue, which we excluded in Table 5, but will return to now. It is not clear why these animals apparently showed more certainty about the presence of a predator than the others. These individuals were perhaps either more (or less) naïve than their counterparts about leopard hunting strategies.

### Black-and-White Colobus Monkeys

Similar to the above examples, Schel et al. (2009, 2010) report on Black-and-White Colobus monkeys (*Colobus polykomos* and *Colobus guereza*) that have calls which fit the two-compartment frame. These monkeys have three types of calls, *snorts*, *roaring* sequences, made of a series of *roars*, and a *snort-roar* sequence. The single snort is typically used for terrestrial predator contexts (not eagles), repetition of roars for leopard and eagle-related situations (with significant structural differences between the two), and the *snort-roar* sequence appears most often related to leopards. For the two-compartment frame, we propose the first compartment contains *snort*, which is never repeated, and the second compartment contains a *roaring* sequence.

### De Brazza’s Monkeys

Bouchet et al. (2012) studied 23 De Brazza monkeys (*Cercopithecus neglectus*) in captivity that included three juvenile males, three juvenile females, five adult males, and 12 adult females, all captive-born. The inclusion of the juvenile monkeys allowed for developmental study of calls, which becomes important for our study. They report that the monkeys produced 10 distinct call types; we will focus on three of them, *On, I,* and *OnI* since the first two together represent the third. Though the De Brazza study described here does not focus on alarm calls, like our other examples, it highlights the ontogeny of a combined call system in an Old World monkey, which is pertinent to our theory that Merge is not necessary for combining two calls.

*On* calls occurred with gazes directed to the adult male by adult females as well as both sexes of juveniles. The adult male made this call when gazing at zoo-keepers, the research observer, or neighboring groups. *I* calls were uttered by juveniles when approaching the adult male to establish physical contact. *OnI* calls were made by adult females and juveniles of both sexes when approaching a male but with ambivalence about whether to approach or escape. The distribution of these calls among juveniles and adults is given below (Table 6).

**Table 6:** Distribution of three call types across age and sex in DeBrazza’s monkeys. Adapted from data in Bouchet et al. (2012).

Females		Males	
Juveniles	Adults	Juveniles	Adults
*On*	*On*	*On*	*On*
*I*		*I*	
*OnI*	*OnI*	*OnI*	

*On* occurs with both juvenile and adult females and males, while *I* occurs only with juveniles of both sexes. *OnI* occurs with female and male juveniles and with female adults.

Let us turn to the question of whether the two-item *OnI* is a product of a combinatorial operation or is learned whole but fit into a dual-compartment frame. Among juveniles of both sexes we find *On, I,* and *OnI*; *OnI* here could be viewed as resulting from a combinatorial process. However, when we look at the adult female, we see a clear indication that *OnI* cannot be the result of an operation that combined *On* and *I*. This is because among females, *On* occurs but *I* does not, yet *OnI* does occur. It is important to note that as juveniles, the females produced both *On* and *I* as well as *OnI*, hence there is presumably awareness that the *OnI* utterance has parts that fit into the whole. Our suggestion is that this fitting the parts into the whole is made possible by the kind of dual-compartment frame we argued for the putty-nosed monkey system. Although *I* is lost in the adult vocal repertoire, presumably the dual-compartment frame structure holds for the adult *OnI*. Crucially, the two-term call *OnI* is not the product of a combinatorial operation such as Merge.

## Concluding Remarks

Previous research showed that there is a fundamental difference between AB combinations and more complex A^n^B^n^ combinations that require hierarchy. Cotton-top tamarins and very young human infants can only compute the simple AB combinations, while humans, after a certain age, can learn the more complex array easily (Fitch and Hauser, 2004; Milne et al., 2016). Experiments by Friederici et al. (2006) showed that there is a neuroanatomical distinction between AB sequences and A^n^B^n^. While the former recruits the frontal operculum, the latter recruits, in addition, the phylogenetically newer Broca’s area. These experiments on tamarins and on human subjects were conducted with artificially created stimuli. We studied the vocal repertoire of Old World monkeys, and found that their calls were limited at most to a combination of two items. We argue this is equivalent to the AB sequence identified earlier using artificial stimuli. What we can deduct from this is that nonhuman primates likely recruit the frontal operculum to create a dual-compartment frame which allows up to two-term calls, but no more, as predicted by previous research. In contrast, humans tap the combinatorial operation of Merge in the Broca’s area to create a potentially infinite array of hierarchical structures. As far as we can tell, there is currently no evidence for Merge in nonhuman primate combined calls.

## Ethics Statement

This study is exempt from ethical requirements as it utilizes previously reported data only. The studies that were reported in the manuscript were carried out in accordance with the recommendations of their respective Animal Care and Research Protocols.

## Author Contributions

SM conceived the paper and performed the analyses. SM and EC researched and interpreted the data and drafted the paper. Both authors contributed to manuscript revision, read, and approved the submitted version.

### Conflict of Interest Statement

The authors declare that the research was conducted in the absence of any commercial or financial relationships that could be construed as a potential conflict of interest.

## Footnotes

^1^In this article, we put forth an incremental approach to the emergence of an infinite, recursive combinatorial system, in line with Townsend et al.’s (2018) observations. Simple cases of compositionality, as seen in primate call combinations, are composed by means of a dual-compartment frame, which may have later served as an input to Merge. However, we do not follow Townsend et al. (2018) in assuming that (frozen) phrasal expressions are structureless (e.g., “duck and cover”), since there is evidence indicating that even simple words comprise a hierarchical structure (see Nobrega and Miyagawa, 2015). Thus, although the dual-compartment frame may have furnished the emergence of Merge, it possibly did not remain active for the formation of linguistic objects, as typically assumed by gradualist approaches (see Progovac, 2015). In our view, Merge —once available— was responsible for the derivation of any linguistic object, from words to sentences.

^2^For discussion about Merge, against, and for see, for example, Everett (2005), Kershenbaum et al. (2014), and Nevins et al. (2009).

^3^A reviewer pointed out the work, Kershenbaum et al. (2014), in which a wide range of animal vocal sequences, from birds to whales to primates, is studied, with the intent of testing to see if these calls, some of which are quite complex, can be described as Markovian vocal calls, which would fit well within the Chomsky hierarchy. Their conclusion is that there are calls that may best be described as non-Markovian. It is interesting that primate calls do not fall into the claimed non-Markovian calls; at this point, our understanding is that primate calls, such as the Old World Monkey calls we have studied, fall within the more traditional view of animal communication, which allows for description by a regular grammar.

^4^Our claim is that the Old World Monkey calls comprise a system that can be described by regular grammar, and one that Friederici et al. (2006) show as using the frontal operculum. This is the same conclusion as Fitch and Hauser (2004), but using the actual vocalization of the animals. This system, and the system in the phylogenetically newer Broca’s area that allows hierarchical structure, together comprise human language. It is possible to view both systems as having existed prior to the formation of human language; language simply tapped these pre-existing resources. In this way, language did not arise from primate alarm calls.

^5^A reviewer wonders how our theory based in part on Merge compares to alternative views such as that of Lieberman (2015, 2016). Lieberman argues that hierarchical syntactic structures cannot be considered the product of a genetically determined, species-specific operation. According to his view, hierarchical structures may be acquired by means of associative motor learning, similarly to learning how to walk. If his assumption is on the right track, one should expect that the type of structural embedding observed in natural languages is arbitrarily determined—a point that is not addressed by the author—even though it is patently uniform across the species. Languages do vary in terms of lexical items and superficial distribution, but they do not vary with respect to the nature of sequences it can generate.
